# Prevalence of mental disorders, suicidal ideation and suicides in the general population before and during the COVID-19 pandemic in Norway: A population-based repeated cross-sectional analysis

**DOI:** 10.1016/j.lanepe.2021.100071

**Published:** 2021-02-27

**Authors:** Ann Kristin Skrindo Knudsen, Kim Stene-Larsen, Kristin Gustavson, Matthew Hotopf, Ronald C. Kessler, Steinar Krokstad, Jens Christoffer Skogen, Simon Øverland, Anne Reneflot

**Affiliations:** a Centre for Disease Burden, Norwegian Institute of Public Health, Zander Kaaes Gate 7, PO Box 973 Sentrum, 5-5808, 5015 Bergen, Norway; b Department of Mental Health and Suicide, Norwegian Institute of Public Health, Marcus Thranes Gate 6, 0473 Oslo, Norway; c PROMENTA Research Center, Department of Psychology, University of Oslo, Blindern, 0317 Oslo, Norway; d Department of Mental Disorders, Norwegian Institute of Public Health, Marcus Thranes Gate 6, 0473 Oslo, Norway; e Department of Psychological Medicine, Institute of Psychiatry, Psychology and Neuroscience, King's College London, 16 De Crespigny Park, Camberwell, London SE5 8 AF, United Kingdom; f Biomedical Research Centre, South London and Maudsley NHS Foundation Trust, 16 De Crespigny Park, Camberwell, London SE5 8 AF, United Kingdom; g Department of Health Care Policy, Harvard Medical School, 180 Longwood Avenue, Boston, MA 02115 United States; h HUNT Research Centre, Department of Public Health and Nursing, Norwegian University of Science and Technology, Forskningsvegen 2, 7600 Levanger, Norway; i Levanger Hospital, Nord-Trøndelag Hospital Trust, Kirkegata 2, 7600 Levanger, Norway; j Department of Health Promotion, Norwegian Institute of Public Health, Zander Kaaes gate 7, 5015 Bergen, Norway; k Center for Alcohol & Drug Research, Stavanger University Hospital, 4010 Stavanger, Norway; l Department of Public Health, Faculty of Health Sciences, University of Stavanger, 4021 Stavanger, Norway

**Keywords:** COVID-19, Mental disorders, Suicidality, Suicides, Prevalence, General population, Epidemiological survey, Diagnostic interview

## Abstract

**Background:**

Self-report data on mental distress indicate a deterioration of population mental health in many countries during the COVID-19 pandemic. A Norwegian epidemiological diagnostic psychiatric interview survey was conducted from January to September 2020, allowing for comparison of mental disorder and suicidal ideation prevalence from before through different pandemic periods. Prevalence of suicide deaths were compared between 2020 and 2014–2018.

**Methods:**

Participants from the Trøndelag Health Study (HUNT) in Trondheim were recruited through repeated probability sampling. Using the Composite International Diagnostic Interview (CIDI 5.0) (*n* = 2154), current prevalence of mental disorders and suicidal ideation was examined in repeated cross-sectional analyzes. Data on suicide deaths was retrieved from the Norwegian Cause of Death Registry and compared for the months March to May in 2014–2018 and 2020.

**Findings:**

Prevalence of current mental disorders decreased significantly from the pre-pandemic period (January 28th to March 11th 2020; 15•3% (95% CI 12•4–18•8)) to the first pandemic period (March 12th – May 31st; 8•7% (6•8–11•0)). Prevalences were similar between the pre-pandemic period and the interim (June 1st July 31st; 14•2% (11•4–17•5)) and second periods (August 1st-September 18th; 11•9% (9•0–15•6)). No significant differences were observed in suicidal ideation or in suicide deaths.

**Interpretation:**

Except for a decrease in mental disorders in the first pandemic period, the findings suggest stable levels of mental disorders, suicidal ideation and suicide deaths during the first six months of the COVID-19 pandemic compared to pre-pandemic levels. Potential methodological and contextual explanations of these findings compared with findings from other studies are discussed.

**Funding:**

None.

**Research in context**

**Evidence before this study**

We searched APA Psychinfo, OVID Medline, Embase, Scopus, and Web of Science with the search terms COVID-19, SARSCOV2, Severe Acute Respiratory Syndrome, mental health, depression, anxiety, suicidal behaviour and drug abuse or variations of these search terms for articles in English published until December 3rd 2020 with no limitation on start date. The search returned 1977 articles describing studies in which the great majority relied on non-probability sampling strategies, focused on population subgroups like health care workers, used non-validated measures to assess mental health outcomes, and lacked a pre-pandemic baseline sample. Only 35 studies used a probability sampling strategy and employed validated screening instruments to examine mental health in the general population. All but five of these reported either high scores or an increase in mental distress during the pandemic. Two of the studies found continuously increased rates of depressive symptoms, but a decrease in anxiety symptoms after initially high scores early in the pandemic. A Dutch study found no increase in either anxiety or depressive symptoms. Only one study used a validated diagnostic scoring tool, and reported a 10% increase in at least one common mental disorder in the first month of the pandemic compared with baseline measures from 2017 in a Czech sample of 3021 participants.

**Added value of this study**

The present study is based on data from a diagnostic interview survey done on probability samples from the general population in the third largest city of Norway, and on data from the nationwide Norwegian Cause of Death Registry. It is the first study to compare prevalence levels of mental disorders defined by diagnostic criteria before and during several periods in the first six months of the COVID-19 pandemic. The study also reports national suicide death rates in the first period of the pandemic, comparing these with the same period in 2014–2018. The richness of the survey data allowed for examination of multiple subgroups defined by sociodemographic and health characteristics. A significantly lower prevalence of current mental disorders was found in both the general population and several subgroups in the first period of the pandemic in Norway compared with pre-pandemic levels. No significant difference was found in prevalence rates between the pre-pandemic period, and the interim period with lower transmission rate and the early part of the second period in Autumn 2020. Suicidal ideation and suicide deaths also showed stable rates during the pandemic compared with pre-pandemic levels.

**Implications of all the available evidence**

The great majority of reviewed studies suggest that the COVID-19 pandemic is a significant threat to public mental health, with strength of reported associations varying from extremely high to moderate. The present study did not confirm such findings. One explanation for the differences in findings may be the higher case-threshold in diagnostic instruments compared to screening instruments. However, the evolvement of the pandemic, as well as policy enactment and measures introduced to counteract this, have varied between settings. This may give context-specific differences in the impact of the pandemic on public mental health in the first six pandemic months. More research based on high quality data in different settings is needed to gain a fuller picture of the pandemic impact on both public mental health, and the mental health of vulnerable subgroups, as the pandemic evolves and in its aftermath.

Alt-text: Unlabelled box

## 1. Introduction

The first coronavirus disease 2019 (COVID-19) case was confirmed in Norway February 26th 2020. The Norwegian government implemented several social distancing measures to contain the spread of the virus on March 12th, and the national epidemic was deemed under control in late April followed by a gradual relaxation of restrictions during the Summer. However, smaller outbreaks led to re-introduction of local measures in early Autumn.

There is a profound concern that the pandemic will give a mental health crisis [1], which has led to a rapid propagation of surveys exploring mental health issues during the pandemic. Most of these have important limitations. Many are online surveys, based on self-selected non-probability or convenience samples with important biases [2]. Most have a cross-sectional design with one-time data-collection early in the pandemic, precluding comparisons of mental health in the study population before and during the pandemic. Only a few high-quality studies based on probability samples have compared mental distress in the general population during the pandemic with pre-pandemic levels. Surveys from the United Kingdom (UK), United States (US), and Czech Republic found dramatic increases in population mental distress in the first pandemic months from baseline measures in 2017–2019 3, 4, 5, 6, 7. In contrast, a Dutch study found no increase in prevalence of anxiety and depressive symptoms in March 2020 compared to March 2019 [8]. Females, young adults, those unemployed or with less economic resources, students, people with no partner or living with young children, and people with pre-existing physical and mental health conditions seemed to have a higher risk of increased mental distress during the pandemic 3, 4, 5, 6.

The published studies have been conducted in the first months of the pandemic, and have exclusively relied on questionnaires or diagnostic screening tools with a short reference-period. Measures of this sort are well-suited to describe an acute surge in mental distress, which may recede as people adjust to the new normal [4]. On the other hand, chronic stressors, such as the anticipated economic recession [9,10], may cause a long-term deterioration of public mental health. However, mental distress is not equivalent to mental disorders, and studies of the latter are needed to examine whether the seemingly deterioration of public mental health during the pandemic is of a magnitude that requires prevention, support and care [11].

The present study has the advantage of stronger data-sources and longer follow-up than previous surveys evaluating the effects of the COVID-19 pandemic on public mental health. Using data from an epidemiological psychiatric diagnostic interview survey that was already ongoing at the time of the COVID-19 outbreak, as well as data from the Norwegian Cause of Death registry (CoDR), the aim of the present study was to compare prevalence of i) current mental disorders, ii) suicidal ideation, and iii) suicide deaths before and during different periods in the first six months of the COVID-19 pandemic in the general adult population and among groups with suspected risk for increased mental health problems during the pandemic.

## 2. Methods

This present report complies with the STROBE statement. The survey is registered at ClinicalTrials.gov (identifier: NCT04661228). The survey was a collaboration between the Norwegian Institute of Public Health (NIPH) and the HUNT Research Center and approved by the Regional Committee for Medical Research Ethics (2017/28/REK midt).

### 2.1. Study design and participants

The psychiatric interview survey was a sub-project in the Trøndelag Health Study (HUNT), aiming to assess prevalence of mental disorders in the general adult population. The HUNT study is a longitudinal, population-based health study, conducted in four independent waves since the 1980s in the mid-Norway counties Nord-Trøndelag and Trøndelag (last wave only). Every county resident is invited to either the Young HUNT (ages 13–19) or HUNT (above age 20) part of the study. Respondents to the psychiatric interview survey were sampled among HUNT participants aged 20 to 65 years from the city of Trondheim. HUNT in Trondheim had a participation rate of 43%, (women: 49.1%, men: 36.5%) [12].

The targeted sample size for the psychiatric survey was 2000 participants, and a sub-sample of 7000 persons from the study population of HUNT participants were invited to reach this goal. The only exclusion criterion was insufficient understanding of Norwegian or English. We had complete lists with postal addresses and mobile phone numbers for all eligible participants as well as information about date of birth and gender. Potential participants were sampled in four draws based on probability sampling over the course of the data-collection period, which lasted from January 28th to September 18th 2020. Each draw selected a random subsample from the study population, which makes it legitimate to compare time-trends. Younger persons were oversampled to adjust for the expected larger nonparticipation among these age-groups [12]. Men were oversampled to constitute 58% of the first drawn sample, however, no gender-specific sampling was done in the three later draws.

### 2.2. Procedures and outcomes

The predesignated participants were informed about the project through postal letters. A week after receipt of the letter, each received an SMS with information on how to sign up for the psychiatric interview. One SMS reminder was sent to persons who did not respond to the initial invitation. Four contact attempts were made to schedule the interview amongst those who registered (three by phone and one final by SMS). All participants received a £25 gift card. Computer assisted face-to-face or telephone interviews were conducted by trained and certified interviewers at a local field station. Due to social distancing measures introduced March 12th 2020, all interviews from March 16th to July 13th were conducted by telephone. From mid-July to mid-September, 188 interviews were conducted face-to-face while 278 interviews were conducted by telephone, allowing us to compare differences in identified cases of mental disorder by mode.

The World Health Organization (WHO) Composite International Diagnostic Interview, fifth version (CIDI 5.0)¸ developed for the WHO World Mental Health (WMH) Surveys [13], was used for the data-collection [14]. CIDI 5.0 is a standardized interview assessing 30-days, 12 months and lifetime prevalence for several mental and substance use disorders according to diagnostic criteria in the Diagnostic and Statistical Manual of Mental Disorders 5th edition (DSM-5) [15] and International Classification of Diseases 10th edition (ICD-10) [13]. It has good concordance with diagnostic instruments such as the Structured Clinical Interview for DSM-IV (SCID) [16] and Schedules for Clinical Assessment in Neuropsychiatry (SCAN) [17].

Two outcome measures were employed in the survey part of this study. *Current mental disorder* was defined as presence of a mental disorder during the 30 days before interview (yes/no). The following mental disorders were included in this variable: major depressive disorder, bipolar type I and II disorders, generalized anxiety disorder, panic disorder, specific phobia, agoraphobia, social anxiety disorder, alcohol use disorder and drug use disorder. Operationalization of diagnoses was based on algorithms developed for CIDI 5.0 in WMH. *Current suicidal ideation* was defined as self-reported presence of suicidal ideation (thoughts of killing oneself or wishing one was dead) during the 30 days before the interview (yes/no). Valid responses are required to progress in the CIDI interview, and there was no missing in these data. However, due to premature interview drop-out, information on presence of substance use disorder was missing for two respondents. As we had valid information on all other diagnoses for these respondents, they could still be included in the binary outcome variables.

**Appendix Fig. 1:**
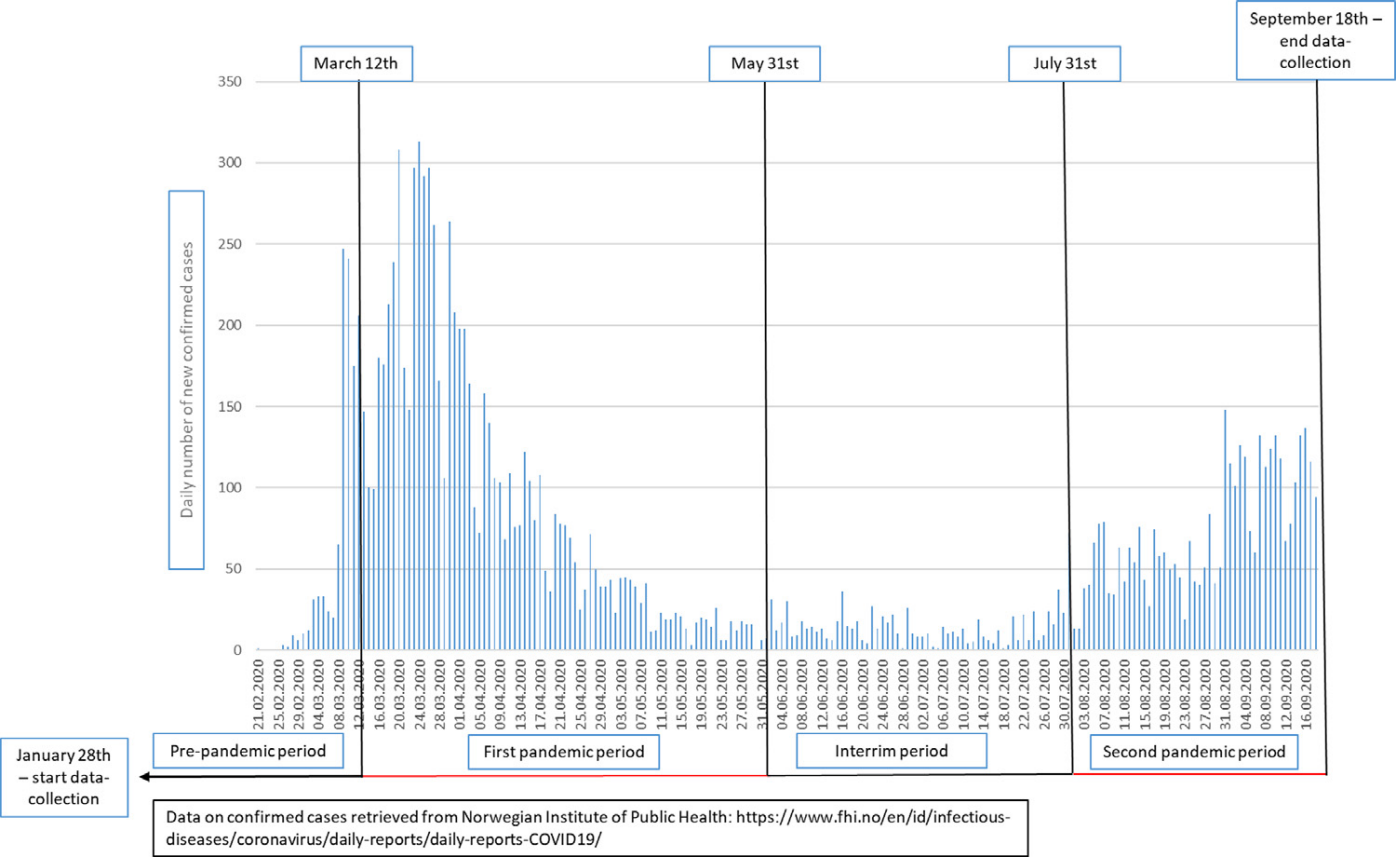
Timeline of daily new confirmed COVID-19 cases in Norway by test-date and categorization of the pandemic periods employed in the current study.

**Appendix Fig. 2:**
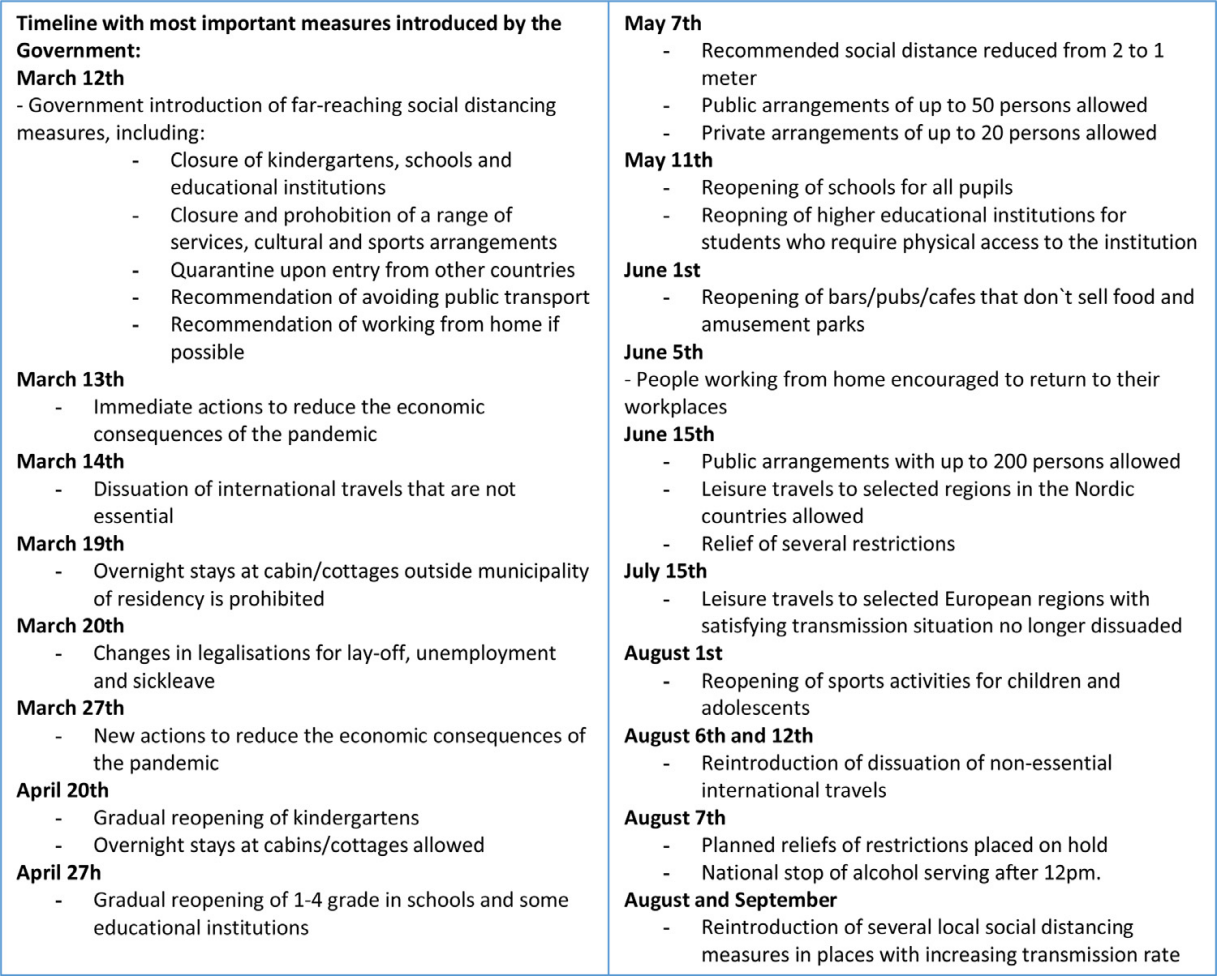
Timeline of most important social distancing measures introduced by the Norwegian Government in the period 21.02.2020 to 18.09.2020. Information retrieved from the official website of the Norwegian Government. https://www.regjeringen.no/no/tema/Koronasituasjonen/tidslinje-koronaviruset/id2692402/.

The primary predictor measure was a four-category variable of *pandemic periods,* defined according to a combination of disease transmission in the population and presence of social distancing measures (see Appendix Figs. 1 and 2 for details). The pre-pandemic period was defined from start of data-collection January 28th to March 11th 2020. The first pandemic period lasted from the date when the majority of national measures were first introduced (March 12th), the same date as the first COVID-19 related death in Norway and WHO declared a global pandemic, to May 31st, when a trend of low transmission rate had stabilized, and several measures were gradually released. The interim period lasted from June 1st to July 31st, and was characterized by low rates of transmission, hospitalizations and deaths, and further release of measures. The second pandemic period was set to start August 1st, indicating a new period with increasing transmission rate and ending with finalizing of the data-collection on September 18th. Participants were categorized to periods based on their interview date. All other variables were based on self-reported information. *Gender* was a binary variable (man/woman) based on reported gender identity. Persons identifying as neither man nor woman were excluded from the analyzes (*n* = 5), as the small number did not allow for meaningful analyzes on this group. *Age-group* was categorized as age 20–29, 30–39, 40–49, and 50–65. *Level of education* was categorized as i) high school level or equivalent, ii) higher education – lower degree (less than four years at a University or equivalent), and iii) higher education – higher degree (more than four years at a University or equivalent). *Living with partner* was coded “yes” if the respondent reported to be married or living with someone in a marriage-like relationship and “no” otherwise. *Age of youngest child in household* was categorized as i) no children or children above 18, ii) youngest child aged 0–5 years and iii) youngest child aged 6–17 years. Information on age of youngest child, based on household information assessed before the onset of the interview, was missing for 90 respondents, and these were excluded from the relevant analyzes. *Physical illness* was defined as a binary variable (yes/no) based on reported presence of health states associated with increased risk of severe COVID-19 infections: severe obesity (BMI≥40), cardiovascular diseases, cancer, chronic lung disease (excluding asthma), diabetes, chronic liver disease or kidney disease, and neurological diseases [18]. The *lifetime mental disorder* variable included lifetime presence of any of the mentioned mental disorders, while *previous mental disorder* included those who had a lifetime history of mental disorder, but who did not satisfy criteria for current mental disorder.

Data on suicide deaths over the three months period March to May for 2014–2018 and 2020 were retrieved from an early release of publicly available statistics from the Norwegian Cause of Death Registry (CoDR). The registry data are based on death certificates, and provides information about the date and cause of death in accordance with the ICD-10 [13]. Roughly half of the underlying causes of death are determined by a semi-automatic coding program (ACME) whereas the other half is coded manually by professional medical coders [19]. The CoDR data normally covers 98% of deaths among Norwegian residents [20]. The early release data from 2020 had a coverage rate of 92%. The completion of the 2020 registration is expected to give an increase in all causes of death, including fatalities that more often go to autopsy, such as suicides.

### 2.3. Statistical analyzes

The survey data were analyzed as repeated cross-sections. First, we constructed weights to adjust for the gender-specific sampling, which were applied to all calculations of proportions and associations. Second, we calculated descriptive characteristics of the total sample and the participants in each of the four pandemic periods. We report unweighted numbers and weighted proportions. We used two-tailed Pearson chi-squared design-based statistics to explore statistical significant differences in characteristics between the samples who participated before and during the pandemic (Appendix Table 2). Third, we examined the weighted proportion with current mental disorder and suicidal ideation in each pandemic period in the total sample as well as stratified by sub-groups defined by sociodemographic or health characteristics. Some groups were collapsed for the analyzes of suicidal ideation. The proportions are presented as percentages with 95% confidence intervals (CI). Statistical significance was evaluated as difference in means at the 0.05 level (*p*-value≤0•05) between the pandemic periods and the pre-pandemic period. The *p*-values were obtained from logistic regression using pre-pandemic period as reference category. Fourth, we explored the risk of having a current mental disorder in each period of the pandemic compared to the pre-pandemic period for the total sample and stratified by groups defined by family or health status, using the four-category pandemic variable as the predictor variable in a logistic regression model. These results are presented as odds-ratios (OR) with 95% CI unadjusted and adjusted for gender, age as a continuous variable and educational categories. Fifth, prevalence of current mental disorders and suicidal ideation in each pandemic period by gender was visualized using bar plots. Finally, we compared numbers and age-adjusted suicide death rates per 100,000 in March-May between the years 2014–2018 and 2020. Stata 15 was used for analyzes.

### 2.4. Role of the funding source

No external funding was given for this study. The corresponding author had full access to all data in the study. Submission was approved by all co-authors. For the purpose of open access, the author has applied a CC BY public copyright licence to any Author Accepted Manuscript (AAM) version arising from this submission.

## 3. Results

**Fig. 1:**
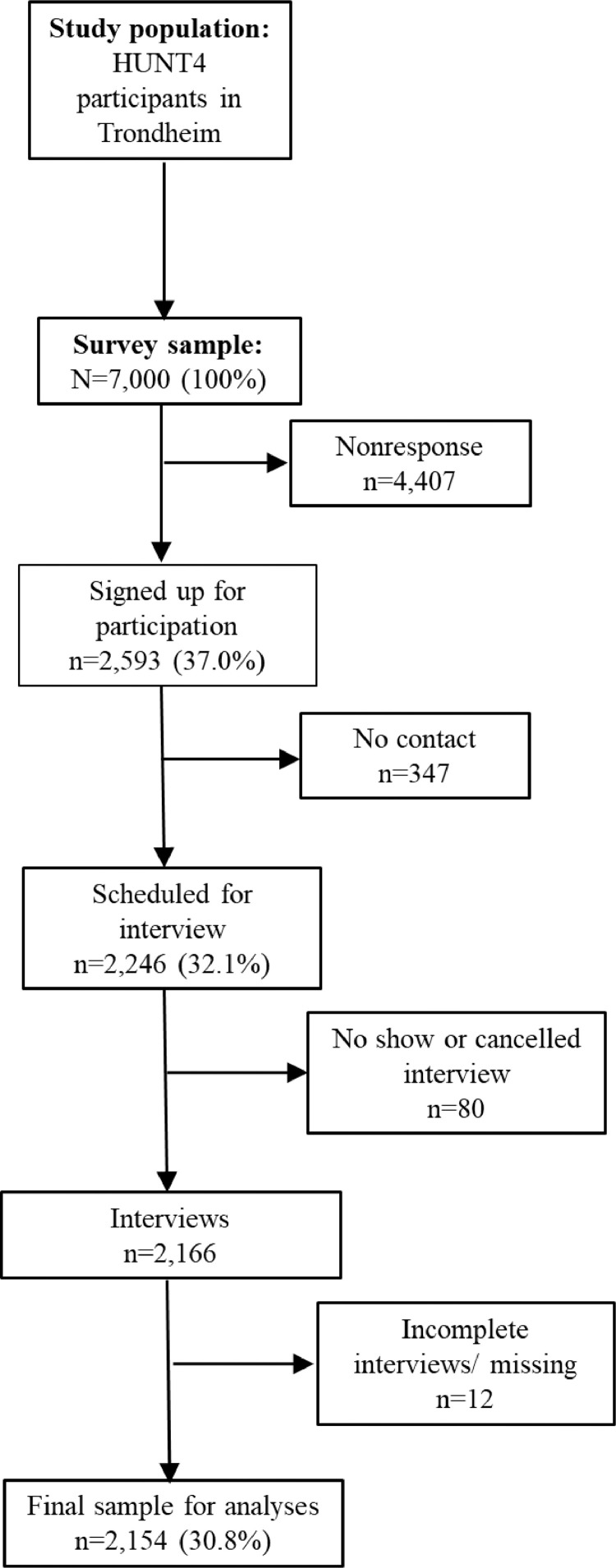
Flow-chart of survey participation process.

Fig. 1 details the participation process. The final participation rate was 30•8% (*n* = 2154), with relatively similar rates across the four pandemic periods (range: 28•7–32•8%; Table 1), and between invitation to face-to-face (29•1%) and telephone interview (34•8%). No statistical difference in prevalence of current mental disorders was found in the period the two modes of data-collection were used simultaneously (face-to-face: 14•4% (95% CI 10•0–20•2), telephone: 12•4% (95% CI 9•0–16•8), *p* = 0•540).

**Table 1:** Sociodemographic characteristics in numbers and weighted^1^ percent of total sample and in each period of the pandemic.

	Total sample	Pre-pandemic period	First period	Interim period	Second period
	*N* (%)	*n* (%)	*n* (%)	*n* (%)	*n* (%)
**Total**	2154 (100)	563 (26•5)	691 (32•1)	530 (24•3)	370 (17•0)
Participation rate (%)	30•8	29•8	32•8	31•4	28•7
**Gender**^2^					
Men	899 (39•2)	322 (43•4)	257 (37•7)	192 (38•8)	128 (35•9)
*Participation rate men (%)*	*27•8*	*28•7*	*28•8*	*26•5*	*25•9*
Women	1255 (60•8)	241 (56•6)	434 (62•3)	338 (61•2)	242 (64•1)
*Participation rate women (%)*	33•3	31•3	35•6	34•7	30•8
**Age**					
Mean (SE)	39•0 (12•6)	38•3 (13•2)	39•4 (12•0)	39•2 (12•8)	39•1 (12•4)
**Age groups**					
20 to 29 years	654 (30•3)	200 (35•8)	187 (26•8)	163 (30•6)	104 (28•1)
30 to 39 years	539 (24•8)	132 (22•4)	176 (25•6)	129 (24•2)	102 (27•6)
40 to 49 years	488 (22•7)	106 (18•9)	189 (27•4)	109 (20•7)	84 (22•7)
50 to 65 years	473 (22•2)	125 (23•0)	139 (20•2)	129 (24•4)	80 (21•6)
**Education**					
High school level	635 (29•7)	203 (36•5)	197 (28•5)	133 (25•3)	102 (27•7)
Higher education, lower degree	719 (33•5)	167 (30•2)	245 (35•5)	184 (34•5)	123 (33•3)
Higher education, higher degree	800 (36•8)	193 (33•3)	249 (36•0)	213 (40•2)	145 (39•0)
**Living with partner**					
Yes	1519 (70•3)	379 (66•5)	515 (74•5)	359 (68•0)	266 (71•9)
No	635 (29•7)	184 (33•5)	176 (25•5)	171 (32•0)	104 (28•1)
**Age of youngest child in household**^3^					
No children	1250 (60•8)	370 (69•4)	367 (55•3)	310 (60•7)	203 (58•1)
0–5 years	360 (17•3)	78 (13•8)	129 (19•5)	82 (16•2)	71 (20•2)
6–17 years	454 (21•9)	93 (16•8)	168 (25•3)	117 (23•1)	76 (21•7)
**Health status**					
Physical illnesses^4^	206 (9•5)	50 (8•8)	63 (9•1)	56 (10•7)	37 (10•0)
Lifetime mental disorder	1112 (51•8)	297 (53•5)	346 (50•2)	272 (51•0)	197 (53•2)
Previous mental disorder^5^	847 (39•3)	215 (38•1)	283 (41•1)	196 (36•7)	153 (41•4)
Current mental disorder	265 (12•5)	82 (15•4)	63 (9•0)	76 (14•3)	44 (11•9)
Current suicidal ideation	79 (3•7)	18 (3•2)	29 (4•2)	17 (3•2)	15 (4•1)

1 Proportions weighted to adjust for gender-specific sampling.

2 Gender identity variable.

3 Age of youngest child missing for 90 respondents.

4 Obesity, cardiovascular diseases, cancer, chronic lung disease (excl. asthma), diabetes, chronic liver disease or kidney disease.

5 Persons with a lifetime history of mental disorder, but no current mental disorder.

Compared to the general population the survey participants were more often females, younger and had higher educational level (Appendix Table 1). Participants during the pandemic had higher education and were more often females, and living with a partner or with preschool children than the pre-pandemic participants, but there was no statistical difference in terms of age (under or above age 40), prevalence of physical illness, lifetime mental disorder or previous mental disorder (Table 1 and Appendix Table 2).

**Table 2:** Prevalence of current mental disorder before and during the COVID-19 pandemic periods in total sample and by sociodemographic characteristics. Weighted^1^ percent with 95% confidence intervals (CI). Bold indicates significant difference from pre-pandemic period.

	Pre-pandemic period	First period	Interim period	Second period
	% (95% CI)	% (95% CI)	% (95% CI)	% (95% CI)
**Total**	15•4 (12•5–18•8)	9•0 (7•1–11•4)***	14•3 (11•5–17•5)	11•9 (9•0–15•6)
**Gender**^2^				
Men	12•2 (9•0–16•3)	6•6 (4•2–10•4)*	13•0 (8•9–18•5)	11•0 (6•6–17•7)
Women	17•8 (13•5–23•2)	10•5 (7•9–13•7)**	15•1 (11•6–19•3)	12•4 (8•8–17•2)
**Age groups**				
20 to 29 years	25•5 (19•6–32•5)	14•2 (9•9–19•9)**	21•5 (15•8–28•5)	20•3 (13•6–29•1)
30 to 39 years	18•9 (12•8–26•9)	10•1 (6•4–15•5)*	10•6 (6•4–17•1)	**8•8 (4•**7–16•1)*
40 to 49 years	7•3 (3•6–14•3)	7•3 (4•4–12•0)	14•8 (9•2–22•8)	10•7 (5•7–19•3)
50 to 65 years	2•8 (1•0–7•4)	3•1 (1•2–8•0)	8•4 (4•7–14•6)	6•2 (2•6–14•0)
**Education**				
High school level	24•9 (19•2–31•7)	13•9 (9•7–19•4)**	23•2 (16•8–31•1)	14•8 (9•1–23•1)*
Higher education, lower degree	10•9 (6•8–17•1)	9•4 (6•4–13•8)	14•6 (10•2–20•5)	12•1 (7•4–19•1)
Higher education, higher degree	9•0 (5•6–14•2)	4•7 (2•7–8•2)	8•3 (5•3–12•9)	9•7 (5•8–15•7)
**Living with partner**				
Yes	10•8 (7•9–14•6)	6•9 (5•0–9•4)*	10•8 (8•0–14•4)	8•7 (5•9–12•8)
No	24•4 (18•5–31•5)	15•3 (10•7–21•4)*	21•7 (16•1–28•5)	20•0 (13•4–28•8)
**Age of youngest child in household**^3^				
No children	18•7 (14•8–23•2)	10•5 (7•8–14•1)**	17•3 (13•5–21•9)	15•8 (11•4–21•5)
0–5 years	10•2 (4•8–20•2)	6•1 (3•1–11•8)	4•7 (1•8–11•9)	7•0 (2•9–15•7)
6–17 years	7•3 (3•4–14•9)	7•0 (4•0–12•0)	12•0 (7•2–19•3)	8•0 (3•6–16•6)
**Health status**				
Physical illnesses^4^	21•9 (11•8–37•0)	12•7 (6•5–23•5)	15•9 (8•5–27•8)	13•5 (5•7–28•6)
Lifetime mental disorders	28•7 (23•7–34•4)	18•0 (14•3–22•4)**	28•0 (23•0–33•6)	22•3 (17•1–28•7)

1 Proportions weighted to adjust for gender-specific sampling.

2 Gender identity variable.

3 Age of youngest child missing for 90 respondents.

4 Obesity, cardiovascular diseases, cancer, chronic lung disease (excl. asthma), diabetes, chronic liver disease or kidney disease Difference from pre-pandemic period: *p ≤ 0.05, **p ≤ 0.01, ***p ≤ 0.001.

**Fig. 2:**
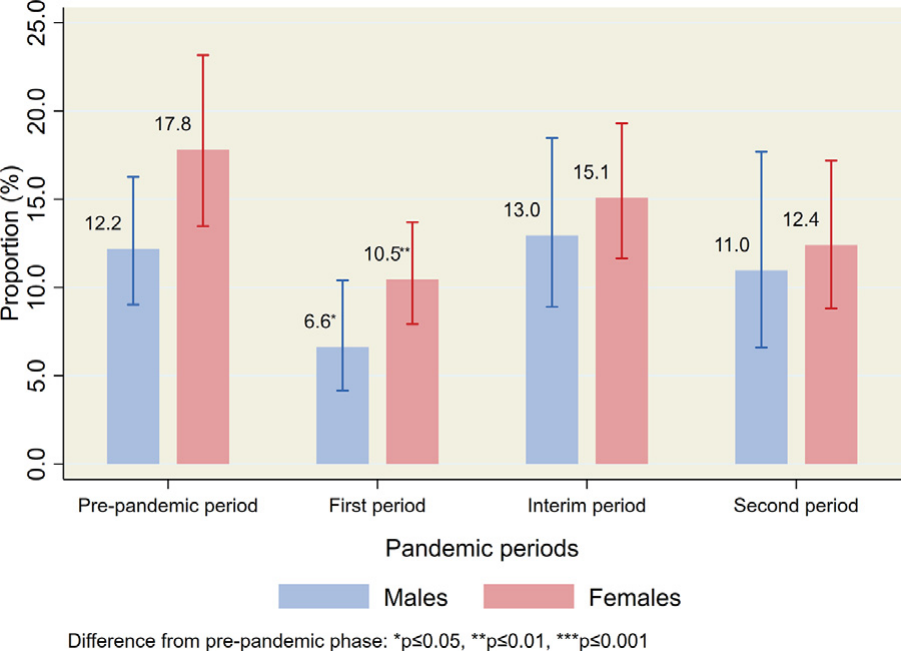
Prevalence of current mental disorders during the different periods of the COVID-19 pandemic by gender. Weighted proportions with 95% confidence intervals.

**Fig. 3:**
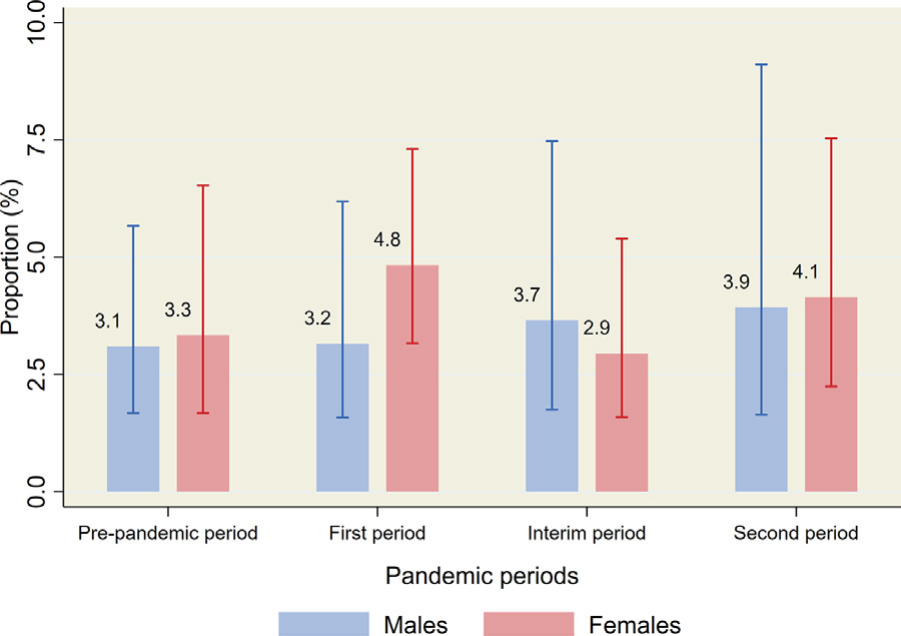
Prevalence of current suicidal ideation during the different periods of the COVID-19 pandemic by gender. Weighted proportions with 95% confidence intervals.

Overall, 15•4% (95% CI 12•5–18•8) of the participants had a current mental disorder in the pre-pandemic period (Table 2). A significant drop in prevalence was observed in the first period of the pandemic in both the total sample (9•0% (95% CI: 7•1–11•4), *p* = 0•001), by gender (Table 2 and Fig. 2), and in several of the sub-groups (Table 2). No significant difference in prevalence was found between the pre-pandemic period and the interim and the second periods in the total sample (interim: 14•3% (95% CI: 11•5–17•5), second period: 11•9% (95% CI: 9•0–15•6), and in fourteen of the sixteen sub-groups examined. Results from the logistic regression analyzes also showed a lower risk of current mental disorder in the first period and no increased risk for current mental disorder in the interim and second periods, in the total samples and among those with lifetime history of mental disorders (Table 3). No significant differences between pre-pandemic and pandemic estimates were found for prevalence of suicidal ideation (pre-pandemic: 3•2% (95% CI: 2•0–5•2); first period: 4•2% (95% CI: 2•9–6•0), interim period: 3•2% (95% CI: 2•0–5•1); second period: 4•1% (95% CI: 2•5–6•6), Tables 3 and 4, and Fig. 3).

**Table 3:** Risk of current mental disorders and suicidal ideation in three periods of the COVID-19 pandemic compared to before the pandemic in total sample^1^ and by family status and health status. Weighted unadjusted and adjusted* odds ratios (OR) with 95% confidence intervals (CI).

	First period		Interim period		Second period	
	OR (95% CI)	*p*-value	OR (95% CI)	*p*-value	OR (95% CI)	*p*-value
**Current mental disorders**						
Total sample						
Unadjusted	0•55 (0•38–0•78)	•001	0•92 (0•65–1•29)	•614	0•74 (0•50–1•11)	•144
Aadjusted*	0•58 (0•41–0•84)	•004	1•02 (0•71–1•45)	•921	0•82 (0•54–1•23)	•329
Not living with partner						
adjustment	0•56 (0•32–0•96)	•034	0•86 (0•52–1•42)	•547	0•78 (0•43–1•41)	•402
Adjusted*	0•59 (0•34–1.02)	•057	0•95 (0•57–1•58)	•837	0•82 (0•45–1•49)	•516
Living with children <age 6^2^						
Unadjusted	0•58 (0•20–1•69)	•316	0•44 (0•12–1•58)	•205	0•66 (0•20–2•22)	•503
Adjusted*	0•55 (0•18–1•69)	•293	0•45 (0•12–1•60)	•215	0•65 (0•19–2•21)	•493
Physical illnesses						
Unadjusted	0•52 (0•18–1•48)	•221	0•67 (0•24–1•88)	•449	0•56 (0•24–1•88)	•337
Adjusted*	0•63 (0•19–2•10)	•452	1•10 (0•70–2•10)	•880	0•63 (0•16–2•41)	•482
Lifetime CMD						
Unadjusted	0•55 (0•37–0•80)	•002	0•96 (0•66–1•40)	•843	0•71 (0•47–1•09)	•120
Adjusted*	0•57 (0•39–0•84)	•004	1•04 (0•70–1•53)	•851	0•77 (0•50–1•19)	•238
**Current suicidal ideation**						
Total sample						
Unadjusted	1•31 (0•71–2•42)	•386	1•00 (0•50–1•98)	•992	1•27 (0•62–2•58)	•511
Adjusted*	1•47 (0•79–2•73)	•226	1•09 (0•54–2•17)	•814	1•40 (0•69–2•87)	•352

⁎ Adjusted for gender, age as continuous variable and education.

1 Due to small numbers, only total sample was examined for suicidal ideation.

2 Age of youngest child missing for 90 respondents.

**Table 4:** Weighted^1^ prevalence of suicidal ideation before and in three periods of the COVID-19 pandemic in total sample and by demographic characteristics. Percent with 95% confidence intervals. Bold indicates significant difference from pre-pandemic period.

	Pre-pandemic period	First period	Interim period	Second period
	% (95% CI)	% (95% CI)	% (95% CI)	% (95% CI)
**Total**	3•2 (2•0–5•2)	4•2 (2•9–6•0)	3•2 (2•0–5•1)	4•1 (2•5–6•6)
**Gender**^2^				
Men	3•1 (1•7–5•7)	3•2 (1•6–6•2)	3•7 (1•8–7•5)	3•9 (1•6–9•1)
Women	3•3 (1•7–6•5)	4•8 (3•2–7•3)	2•9 (1•6–5•4)	4•1 (2•2–7•5)
**Age groups**				
< 40	5•2 (3•2–8•3)	5•5 (3•6–8•4)	5•2 (3•1–8•4)	4•4 (2•3–8•3)
> 40	0•6 (0•1–3•9)	2•7 (1•4–5•2)	0•9 (0•2–3•4)	3•6 (1•6–7•8)
**Education**				
High school level	5•0 (2•6–9•5)	6•7 (3•9–11•2)	3•0 (1•1–7•8)	3•9 (1•5–9•9)
Higher education	2•2 (1•1–4•3)	3•2 (2•0–5•2)	3•3 (1•9–5•6)	4•1 (2•3–7•3)
**Living with partner**				
Yes	2•0 (0•9–4•4)	2•7 (1•6–4•5)	2•0 (1•0–4•2)	1•9 (0•8–4•4)
No	5•7 (3•2–10•0)	8•6 (5•3–13•9)	5•8 (3•1–10•4)	9•7 (5•3–17•1)
**Age of youngest child in household**^3^				
No children	4•1 (2•5–6•8)	5•2 (3•3–8•0)	4•2 (2•5–7•1)	6•4 (3•8–10•7)
0–5 years	1•8 (0•3–11•6)	3•1 (1•1–7•9)	No obs	No obs
6–17 years	1•5 (0•2–9•7)	2•4 (0•9–6•2)	2•5 (0•8–7•6)	1•4 (0•2–9•0)
**Health status**				
Physical illnesses^4^	4•2 (1•0–16•1)	4•8 (1•6–13•9)	3•4 (0•9–12•7)	2•6 (0•4–16•6)
Lifetime mental disorder	5•8 (3•6–9•3)	7•0 (4•7–10•2)	5•9 (3•7–9•5)	6•1 (3•5–10•4)
Previous mental disorder^5^	4•3 (2•3–8•1)	4•6 (2•7–7•8)	3•1 (1•4–6•8)	3•2 (1•4–7•6)
Current mental disorder	9•4 (4•5–18•7)	17•5 (10•0–29•0)	13•1 (7•2–22•6)	16•0 (7•8–29•9)

1 Proportions weighted to adjust for gender-specific sampling.

2 Gender identity variable.

3 Age of youngest child missing for 90 respondents.

4 Obesity, cardiovascular diseases, cancer, chronic lung disease (excl. asthma), diabetes, chronic liver disease or kidney disease.

5 Current mental disorder excluded. *p ≤ 0.05, **p ≤ 0.01, ***p ≤ 0.001.

In March to May 2020, 140 suicide deaths were recorded, equivalent to an age-adjusted suicide rate of 2•8 per 100,000 (Table 5). This does not differ significantly from the average number of 165 suicides for these months in 2014 to 2018 (age-adjusted rate: 3•3 per 100,000 (95% CI: 2•5–4•0)).

**Table 5:** Number age-adjusted rates per 100,000 of suicide deaths for the Norwegian population in the period March to May for the years 2014–2018 and 2020. Data from the Norwegian Cause of Death Registry.

	2014	2015	2016	2017	2018	Average 2014–2018	2020
Rate per 100,000*	3•2	2•9	3•1	3•2	4•1	3.3	2•8
Number of suicides	158	144	155	162	206	165	140

Note:

⁎ Age-adjusted EUROSTAT standard population.

## 4. Discussion

This is the first study to compare mental disorder and suicidal ideation prevalence before and during several periods in the first six months of the COVID-19 pandemic using diagnostic data from the same cohort. The results suggested a significant decrease in current mental disorders in the first period of the pandemic in Norway, with no significant difference between the interim and second period compared with the pre-pandemic period. This trend was also observed in most of the sub-groups examined. No difference was detected in levels of suicidal ideation and suicide deaths before and during the pandemic. The findings are in contrast to studies from the US, UK and Czech Republic, all of which found increase in mental distress in the first pandemic period 3, 4, 5, 6, 7.

Like other studies comparing mental health before and during the COVID-19 pandemic 3, 4, 5, 6, 7 respondents were recruited from a panel of previous survey participants, and as is commonly the case in epidemiological surveys, response rates varied with gender, age and educational level. Individuals with mental health problems, including suicidality, are generally under-represented in health surveys [21]. Mental health may have affected both overall and period-specific participation. However, we found no difference in prevalence of previous mental disorders across periods, indicating relatively similar “baseline” mental health between samples. Participation rates were stable between periods, and the overall time-trends in current prevalence estimates were also observed in the sub-group and adjusted regression analyzes. Thus, while mental health selection may potentially bias the specific prevalence *rates*, this should have less effect on the *associations* between pandemic periods and current mental disorder [22]. Still, the potential of selection bias due to mental health and socio-demographic characteristics should be considered for the internal and external validity of the findings.

The most important difference between the present study and others are in the measures of mental health. Diagnostic instruments assessing mental disorders, like CIDI, also include criteria of functional loss, disability and duration of symptoms that elevate case-finding thresholds compared to scoring tools and questionnaires, which primarily measures shorter periods (ie. two weeks) with psychiatric symptoms or mental distress [23,24]. While the latter may be sensitive to short-term mental health deterioration during the pandemic, this is not equivalent to an increase in mental disorders of clinical relevance.

The COVID-19 pandemic has profound consequences for individuals and societies, but the psychosocial impact may vary between settings. To date, Norway has had a lower rate of transmission, hospitalizations and COVID-19 related deaths than most high-income countries [25], and countermeasures have been milder and of shorter duration. Norway has a strong welfare system, with universal free access to healthcare and economic compensation of sick-leave and job loss. Several rounds of monetary support have aimed to reduce the economic impact of the pandemic. Thus the Norwegian context and policy enactment may have so far curbed a potential public mental health deterioration [26]. These factors may affect the generalizability of the present findings. Future cross-national analyzes should compare the impact of policy enactment, and health and political systems on the psychosocial consequences of the pandemic.

The seeming drop in prevalence of mental disorders in the first pandemic period is somewhat counter-intuitive. The pandemic periods coincided with different seasons, and seasonality could play a part. However, the pattern in seasonality remains unclear [27], with Norwegian examples suggesting higher rates of psychiatric hospital admissions and suicides in the Spring, which is in contrast to our findings [28]. Lower incidence of health outcomes such as myocardial infarction [29] has been found during the first months of the pandemic compared to pre-pandemic levels, and reduced stress has been suggested as one explanation for these findings [29]. This explanation may also apply to mental health outcomes. Further, mental health promoting factors and resilience may also be present in this extraordinary situation, and should be researched to gain a full understanding of the mental health consequences of the COVID-19 pandemic [30].

The strengths of the study include the use of repeated probability samples across several periods before and during the COVID-19 pandemic, the use of diagnostic outcome data of clinical relevance assessed through standardized and validated instruments, and the inclusion of high quality and updated data on suicide deaths from an official registry covering the entire Norwegian population. However, the study also holds important limitations beyond the methodological issues discussed above. The shift from face-to-face to telephone interviews co-occurred with the pandemic onset. Although we found little difference in participation and prevalence estimates between these two interview-modes, we cannot exclude that detection of diagnoses may have differed, particularly in the early phase of telephone interviews when the interviewers were less experienced with this mode. The analyzes are in essence cross-sectional, and we have not measured individual changes in mental disorders and suicidality. The samples may have been underpowered to detect some sub-group differences between the pandemic periods. Further, our decision to base our conclusion of significant difference at the *p*-value ≤0.05 meant that confidence intervals did overlap for some estimates. We did not have information about respondent ethnicity, and the number of mental disorders included was limited. A potential delay in the 2020 CoDR registration of death codes retrieved from autopsies may give a later increase in fatalities ascribed to suicides. Based on the current estimates it is unlikely that additional data will give a higher suicide rate for the 2020 period covered in this study than the average rate observed in 2014–2018, but these results should be interpreted with caution until registration is finalized.

To conclude, the results suggest generally stable levels of current mental disorders and suicidality in the first six months of COVID-19 pandemic in Norway, compared with pre-pandemic levels. However, it is important to consider that the data-collection ended in mid-September 2020. Norway experienced a rapid increase in new COVID-19 cases from late September [25], followed by national re-introduction of stronger social distancing measures. The strain of the population after months of uncertainty, restrictions, and an aggravated economic situation may push more individuals from symptomatic responses to mental disorders. Repeated high quality studies are still needed to closely monitor the public mental health situation during and after the COVID-19 pandemic.

## Author Contributions

Ann Kristin Skrindo Knudsen (AKSK), Anne Reneflot (AR), Simon Øverland (SØ), Steinar Krokstad (SK), and Ronald C. Kessler (RCK) contributed with initiation, planning and design of the survey data-collection. AR and AKSK led the data-collection, and Kristin Gustavson (KG) and Kim Stene-Larsen (KSL) contributed to the progress and quality control of this. AKSK initiated, planned, designed and coordinated the present study. She also conducted the statistical analyzes on the survey data, and led the writing of the manuscript. KSL conducted the literature review, analyzed the registry data and wrote the text for these analyzes. Jens Christoffer Skogen (JCS) produced the figures. AR contributed to the design of the study, in the literature review and verifying the underlying data. All authors, including KG, Matthew Hotopf (MH), RCK, SK and SØ, contributed with input on design and analytical plan, interpretation of results, writing of the first draft, and critical revision of the manuscript and analyzes. All authors approved the submission.

## Data availability

Norwegian data protection regulations and GDPR impose restrictions on sharing of individual participant data. However, researchers may gain access to survey participant data by contacting the publication committee (anne.reneflot@fhi.no). Approval from the Norwegian Regional Committee for Medical and Health Research Ethics (https://helseforskning.etikkom.no) is a pre-requirement for access to the data. The dataset is administrated by the HUNT databank, and guidelines for access to data are found at https://www.ntnu.edu/hunt/data. The study protocol and the informed consent form are available on the two homepages of the project; https://www.ntnu.no/hunt/trondelag/psykisk and https://www.fhi.no/cristin-prosjekter/aktiv/diagnosebasert-undersokelse-psykiske-lidelser-og-ruslidelser/. Analytic codes for the analyzes are available upon request to the corresponding author. The data from the Cause of Death registry are publicly available at http://statistikkbank.fhi.no/dar/.

## Declaration of Interests

Dr. Hotopf reports grants from European Commission IMI/EFPIA, grants from National Institute of Health Research, grants from Medical Research Council, and grants from Economic and Social Research Council outside the submitted work. In the past 3 years, Dr. Kessler was a consultant for Datastat, Inc., Sage Pharmaceuticals, and Takeda. The authors Dr. Knudsen, Dr. Gustavson, Dr. Krokstad, Dr. Skogen, Dr. Stene-Larsen, Dr. Øverland and Dr. Reneflot report no conflict of interest.

## Appendix Supplementary materials

Image, application 1
