# Supplementary material for: Prevalence of mental disorders, suicidal ideation and suicides in the general population before and during the COVID-19 pandemic in Norway: A population-based repeated cross-sectional analysis
Source: Lancet Reg Health Eur. 2021 Feb 27;4:100071. doi: 10.1016/j.lanepe.2021.100071 (PMC8454837; doi:10.1016/j.lanepe.2021.100071)
Supplement: Supplementary file 1 [file mmc1.docx]

Translated abstract - Norwegian

Sammendrag

Bakgrunn

I flere land har data basert på selv-rapport indikert en forverring av befolkningens psykiske helse under COVID-19 pandemien. En epidemiologisk og diagnosebasert psykiatrisk intervju-undersøkelse ble gjennomført i Trondheim fra januar til september 2020. Dette gjorde det mulig å sammenlikne forekomsten av psykiske lidelser og suisidalitetstanker før og under de første periodene i pandemien. I tillegg ble forekomsten av selvmord mellom 2020 og 2014-2018 sammenliknet.

Metode

Deltakere fra Helseundersøkelsen i Trøndelag (HUNT) i Trondheim ble rekruttert via repetert sannsynlighetsutvelging, og intervjuet med Composite International Diagnostic Interview (CIDI, n=2,154). Forekomst av psykiske lidelser og suisidalitetstanker de siste 30 dagene ble sammenliknet i repeterte kryss-seksjonelle analyser. Informasjon om selvmord ble hentet fra Dødsårsaksregisteret, og sammenliknet for månedene mars-mai i 2014-2018 og 2020.

Funn

Forekomsten av psykiske lidelser var signifikant lavere i den første perioden av pandemien (12. mars til 31. mai; 8·7% [6·8-11·0]) sammenliknet med perioden før pandemien (28. januar til 11. mars 2020; 15·3% [95% CI 12·4-18·8]). Forekomsten var lik mellom før-pandemi perioden og mellomperioden (1. juni – 31. juli; 14·2% [11·4-17·5]) og andre pandemiperiode (1. august -18. september; 11·9% [9·0-15·6]). Det ble ikke funnet signifikante forskjeller i forekomst av suisidalitetstanker og selvmord før og under pandemien.

Fortolkning

Med unntak av en nedgang i psykiske lidelser i den første pandemiperioden antyder funnene stabile nivåer av psykiske lidelser, suisidalitetstanker og selvmord i befolkningen før og under de første seks månedene av COVID-19 pandemien. Mulige metodologiske og kontekstuelle forklaringer på funnene sammenliknet med funn fra andre studier blir diskutert.

**Appendix**

**Appendix table 1.** Distribution of age and gender in the population aged 20 to 65 in Norway^1^, the population aged 20 to 65 in Trondheim^2^, the drawn sample to psychiatric interview survey^3^ and among participants in the survey^4^; and participation rate in the survey by age gender and age-group^5^.

|  | **Norway population^1^** | **Trondheim population^2^** | **Total drawn sample^3^**  **N=7,000** | **Participants**  **Trondheim^4^**  **N=2,154** | **Participation rate^5^** |
| --- | --- | --- | --- | --- | --- |
| **Gender** |  |  |  |  |  |
| Men | 51.2% | 51.8% | 46.2% | 41.7% | 27.8% |
| Women | 48.8% | 48.2% | 53.8% | 58.3% | 33.3% |
| **Age-group** |  |  |  |  |  |
| 20 to 29 years | 22.6% | 29.2% | 33% | 30.4% | 28.5% |
| 30 to 39 years | 22.7% | 23.7% | 26% | 25.0% | 29.7% |
| 40 to 49 years | 23.1% | 20.6% | 23% | 22.7% | 30.3% |
| 50+ to 65 years | 31.6% | 26.5% | 18% | 22.0% | 37.5% |
| **Education level** |  |  |  |  |  |
| High school level | 60.0% | 55.1% | - | 29.5% |  |
| Higher education, lower degree | 27.9% | 28.1% | - | 33.4% |  |
| Higher education, higher degree | 12.1% | 16.8% | - | 37.1% |  |

**Appendix table 2.** Demographic characteristics of samples before and during the pandemic, with chi-statistics and absolute difference. Unweighted numbers and weighted proportions.

|  | **Total sample** | **Before pandemic** | **During pandemic** | **Chi-statistics** | **Difference** | |
| --- | --- | --- | --- | --- | --- | --- |
|  | **N (%)** | **n (%)** | **n (%)** |  | **Absolute^1^** | **Relative^2^** |
| **Gender^1^** |  |  |  | Χ^2^(1)= 5.91, p=.016 | 5.8 | 1.1 |
| Men | 899 (39.2) | 322 (43.4) | 577 (37.7) |  |  |  |
| Women | 1,255 (60.8) | 241 (56.6) | 1,014 (62.4) |  |  |  |
| **Age** |  |  |  | Χ^2^(1)= 2.95, p=.098 | -4.1 | 0.9 |
| Under 40 | 1,193 (55.1) | 332 (58.1) | 861 (54.0) |  |  |  |
| Over 40 | 730 (44.9) | 231 (41.9) | 730 (46.0) |  |  |  |
| **Education** |  |  |  | Χ^2^(1)= 16.97, p<.001 | -9.1 | 0.8 |
| High school level | 635 (29.7) | 203 (36.4) | 432 (27.3) |  |  |  |
| Higher education | 1,519 (70.3) | 360 (63.6) | 1,159 (72.7) |  |  |  |
| **Living with partner** |  |  |  | Χ^2^(1)= 5.51, p=.024 | -5.2 | 0.8 |
| Yes | 1,519 (70.3) | 379 (66.5) | 1,140 (71.7) |  |  |  |
| No | 635 (29.7) | 184 (33.5) | 451 (28.3) |  |  |  |
| **Living with preschool children** |  |  |  | Χ^2^(1)= 6.39, p=.013 | 4.7 | 1.3 |
| Yes | 360 (17.3) | 78 (13.8) | 282 (18.5) |  |  |  |
| No | 1,704 (82.7) | 463 (86.2) | 1,241 (81.5) |  |  |  |
| **Health status** |  |  |  |  |  |  |
| Physical illness | 206 (9.5) | 50 (8.8) | 156 (9.8) | Χ^2^(1)= 0.56, p=.466 | -1.0 | 0.9 |
| Lifetime mental disorder^3^ | 1,111 (51.7) | 297 (53.5) | 814 (51.1) | Χ^2^(1)= 0.96, p=.343 | -2.4 | 1.0 |
| Previous mental disorder^4^ | 846 (39.2) | 215 (38.1) | 631 (39.7) | Χ^2^(1)= 0.43, p=.527 | 1.6 | 1.0 |

^1^Difference in percentage points between before and during the pandemic. ^2^Difference in rate points between before and during the pandemic. ^3^Current mental disorder included. ^4^Current mental disorder excluded.
